# Supplementary material for: Effects of cleft lip on visual scanning and neural processing of infant faces
Source: PLoS One. 2024 Mar 27;19(3):e0300673. doi: 10.1371/journal.pone.0300673 (PMC10971660; doi:10.1371/journal.pone.0300673)
Supplement: Attachment — Submitted filename: PLOS ONE Effects of Cleft Lip.docx [file pone.0300673.s001.docx]

**PLOS ONE Effects of Cleft Lip on Visual Scanning and Neural Processing of Infant Faces**

**Abstract**

I would like to see some actual results reported in the abstract - mean values, P values

**Introduction**

I think the introduction wold benefit from more information on EEG – what it is, how it works, how it has previously been used in similar research etc. for those relatively unfamiliar with the details

**93 – 109 –** you need a bit here to explain what ERPs are and give examples to guide readers unfamiliar with the topic

**110 – 118** – this is a nine-line sentence that is very hard to follow and understand. This needs to be broken down into three/four shorter sentences and should be reworded for clarity.

**118** – give an example of LPP for clarity

**127 – 130** – this sentence is hard to follow – I would split into two shorter sentences and restructure

**143 – 154** – are there any ethical considerations here e.g. permission to use images required/not required?

**Methods –** there is no section on statistical analyses used – please include. I see some reference to statistical tests in the results section but these need to be clearly outlined in the methods section e.g. ‘a t-test was used to compare X and Y’ etc. For example, 237 – the analysis used here has not been specified

**237** - Standard deviation would typically be denoted by the ‘±’ symbol

**251 –** State the new P value to be reached following Bonferroni correction for all cases where it was applied

**252** – ‘the interaction resulted because...’ does not make sense – reword

**280** – this is the first time ‘inverted faces’ s referred to – need to explain earlier on in the manuscript

**Discussion**

**375 onwards** - I think a lot of this discussion would be useful to include in the introduction as it explains why you looked at P200, P170 etc – explain these in the introduction to build the rationale. They can then be discussed in less detail in the discussion section as they have already been explained more generally
