# Supplementary material for: Effects of cleft lip on visual scanning and neural processing of infant faces
Source: PLoS One. 2024 Mar 27;19(3):e0300673. doi: 10.1371/journal.pone.0300673 (PMC10971660; doi:10.1371/journal.pone.0300673)
Supplement: Attachment — Submitted filename: Response to Reviewers.docx [file pone.0300673.s002.docx]

Dear Dr Cray,

Thank you for inviting us to revise our manuscript “Effects of Cleft Lip on Visual Scanning and Neural Processing of Infant Faces” with manuscript number PONE-D-23-31387. Please see below for the editor and reviewers’ comments as well as our responses.

Thank you,

Amanda Hahn & KJ Jantzen

**Journal Requirements/Editor’s Comments:**

1. The editor asked that we confirm our manuscript meets PLOS ONE formatting requirements.

The revised manuscript meets the PLOS formatting requirements.

2. The editor noted that PLOS recommends that authors include open data.

All data and analysis code for the current manuscript are publicly available at <https://osf.io/6zm9d/>

3. The editor noted that our manuscript contains figures that may require copyright permissions (figures 1, 5, 6, 7).

All figures flagged were created by the authors themselves for this manuscript. These figures have not been previously copyrighted. Although figure 1 appears to contain identifying information (a facial image), it is actually a composite image of several individual identities and thus reflects a fictional person and does not contain identifying information. The use of a composite facial image for illustrative purposes is noted in the figure caption.

**Reviewer #1 Comments**

1. Reviewer 1 asked that we describe the stimuli used in more detail across the EEG and eye tracking components of the study. They also asked whether a chin rest was utilized to maintain a consistent viewing distance.

Although the original manuscript contained information about the stimulus size and use of a chin rest, we have edited this text for clarity.

Edited text (line 183): “The faces were presented in full color at 600x800px for all elements of the study.”

Edited text (lines 203-205): “Head movement was limited via a chin rest (Tobii) located 85cm from the computer screen (26” DELL monitor) for the duration of the study.”

2. Reviewer 1 asked that we provide a definition of infant cuteness in the introduction.

We now include additional details describing what cuteness is and why perceived cuteness in infant faces may be relevant to caretaking in the introduction of the manuscript.

Added text (lines 58-61): “Because infant cuteness reflects baby-typical features (e.g., large eyes, chubby cheeks, small chin, etc.) that act as an innate releasing mechanism for caretaking responses (Lorenz, 1943), reduced perceptual cuteness may negatively impact parental responses to these infants.”

3. Reviewer 1 asked that we incorporate some discussion of what would be expected with inverted faces for visual scanning patterns.

We thank the reviewer for directing us to the recently published Kuraguchi & Nittono paper, which came out after we had completed the original manuscript. It appears from this paper that inversion may impact cuteness perception in a task-dependent fashion – inversion did not alter the difference scores for perceptual ratings of high vs low cute infants, but it did reduce sensitivity to cuteness in the 2AFC task. The question of whether infant face perception, and/or cuteness perception, relies on more featural (elemental feature) processing or configural (holistic) processing remains an interesting question for future research. As Kuraguchi and Nittono note in the paper, there is some evidence to support the idea that cuteness perception relies on more elemental or featural processing. Other work on the own-race bias in face processing, however, has provided equivocal evidence for the use of configural processing for infant faces (e.g., Hodsoll et al., 2010; Martinez et al., 2020; Proverbio et al., 2011, 2019). We have added a brief discussion of this issue to the revised manuscript.

Added text (lines 532-536): “Interestingly, recent work has suggested that inversion impacts cuteness perception in a task-dependent fashion (Kuraguchi & Nittono, 2023); it remains unknown whether infant face processing relies on configural processing to the same degree as adult face processing.”

Visual scanning patterns for inverted faces were, perhaps unfortunately, not measured in the current study. It is an interesting topic for future work. Based on eye tracking studies using adult faces, it is possible to speculate about what might be observed for infant faces generally. Hills et al. (2013) observed no differences in the number of fixations made to the mouth region for upright versus inverted faces. They did, however, find that inverting adult faces reduced the overall number of fixations made to the eye region. Barton et al., (2006) found that the first fixation made to an inverted face is typically the mouth region, as opposed to the typical first fixation to the eyes for upright faces (e.g., Tyler & Chen, 2006). Taken together, these findings for adult faces suggest that time to first fixation at the eyes would have likely been reduced for inverted infant faces as compared to upright infant faces, but time to first fixation at the mouth would have likely been unaffected by inversion. Whether the impact of inversion on attentional capture by the eyes is similar for cleft vs healthy infant faces remains an open question.

4. Reviewer 1 asked for additional clarity regarding the unit of measurement for the eye tracking study and definitions for the eye tracking measures.

We have updated the results text throughout to include the units of measurement for the eye tracking (seconds) and ERP (uV) data. We have also updated the methods section to provide a more detailed description of the time to first fixation and duration of first fixation. Additionally, we would like to note that we double checked our data in light of the reviewer’s feedback on fixation times and verified the fixation onset times reported in the manuscript are correct.

Added/edited text (lines 301-306): “To evaluate the impact of the cleft lip on visual attention, we calculated the time to first fixation (in seconds) and duration of the first fixation (in seconds) for both the eye and mouth regions of interest. Time to first fixation was defined as the onset of the first gaze fixation in each ROI. The duration of first fixation was the dwell time (or length, in seconds) of the first fixation in each ROI. Fixations were determined using Gazepoint’s internal fixation filter. We utilized these two measures because the provide insight into how quickly and strongly the mouth region of cleft lip/palate infants capture attention in viewers.”

5. Reviewer 1 asked that we provide a rationale for the sample size of our study.

We have included more detail regarding the determination of our final sample size.

Added text (lines 157-159): “Recruitment began May 1^st^, 2022 and concluded June 1^st^, 2022; the sample size reflects the maximum number of participants we were able to recruit during this time period (limited by access to the EEG equipment).”

6. Reviewer 1 asked that we include additional discussion about the generalizability of the results using a non-parent sample when reflecting on parent-child interactions.

We thank the reviewer for this suggestion and believe that incorporating a discussion of the use of parent vs non-parent samples in the literature has improved our manuscript.

Added text (lines 579-604): “Additionally, it is worth noting that this study was conducted on a non-parent sample. The use of nulliparous individuals is common in studies exploring adults’ neural and behavioral responses to infants, however (e.g., Almanza-Sepúlveda et al., 2018; Bos et al., 2018; De Carli et al., 2017; Golle et al., 2015; Esposito et al., 2015; Hahn et al., 2015a, 2015b; Lobmaier et al., 2010; Montoya et al., 2012; Parsons et al., 2011, 2013; Power et al., 1982; Volk et al., 2002, 2005; Zhang et al., 2022). Infant faces are highly biologically-relevant stimuli that have been found to capture adult attention (Thompson Booth et al., 2014) and elicit positive affective responses (Senese et al., 2013) in both parents and non-parents. For example, Thompson Booth and colleagues (2014) found that infant faces preferentially engage visual attention relative to adult faces in both mothers and non-mothers (although this effect was stronger in mothers). Neuroimaging work has similarly demonstrated that infant cues (both facial and vocal) capture attention and elicit reward-related neural activity (relative to adult cues) even among non-parents (reviewed in Ferrey et al., 2016). Results from ERP studies, however, are more mixed – some studies have found differences in the N170 (Weisman et al., 2012) and LPP (Proverbio et al., 2006) responses to infant faces among parents compared to non-parents, while others have not (Noll et al., 2012; Peltola et al., 2014). A recent meta-analysis indicates that these parental status effects may hold true for the N170 (with slightly larger N170 responses among parents compared to non-parents) but not the LPP (Vuorianen et al., 2022). While it is possible that parental status may impact the responses observed here, Schaller (2018) has recently argued that humans have evolved a *parental care motivation system* reflecting psychological mechanisms that have evolved to promote caretaking behavior and parental “instincts” in parents and non-parents alike. Future studies may explore the impact of parental status on adults’ responses to cleft lip/palate (or other facial malformations) to more directly address the impact these have on parent-child interactions.”

7. Reviewer 1 asked that we provide details regarding the interstimulus intervals.

In our original draft, we used the term “intertrial intervals” in the ERP section. We have updated this to read as “interstimulus intervals” to clarify this issue.

Edited text (line 238): “Interstimulus intervals varied randomly between 1000-1500ms.”

We have also added text to clarify that during the eye tracking paradigm, the rating task occurred for each face following the eye tracking measurement. Thus, the interstimulus intervals for the eye tracking study were determined by the time it took to register a cuteness rating.

Edited text for cuteness rating methods (lines 212-213): “Participants advanced to the next task after making their ratings (i.e., there was no time constraint on the rating task).”

Edited text for eye tracking methods (lines 221-222): “After the 10 second viewing period, the rating task described above appeared on screen before advancing to the next trial.”

8. Reviewer 1 asked for more detail regarding the instructions provided to the participants for the ERP study.

Additional details regarding the procedure explained to participants are now included in the revised manuscript.

Edited text (lines 231-235): “During EEG recording, participants were instructed to view the faces presented on the screen (i.e., a passive viewing task). They were asked to focus on the fixation cross that preceded each face and try not to blink when faces were presented. They were also informed that they would be given a break during the viewing task to blink freely and rest.”

9. Reviewer 1 noted three typos in the manuscript (original MS lines 213, 228, and 312).

We thank the reviewer for their careful review of the manuscript. These typos have now been corrected.

**Reviewer #2**

1. Reviewer 2 requested for some results to be reported in the abstract.

We have included the mean difference and p-value for the cuteness perception result in the updated manuscript. Because we summarize the results for the eye tracking and ERP data across multiple measures/components, it is difficult to incorporate the large number of means/p-values for these measures. As such, we have not added these to the revised abstract, however we would be happy to reconsider this decision if the reviewer feels strongly that additional results/statistics be incorporated.

Added text (lines 13-14): “Infants with cleft palate were also rated significantly less cute than their healthy counterparts (mean difference = .73, p<.001).”

2. Reviewer 2 requested additional information about EEG to be included in the introduction for readers who are not familiar with this technique.

Added text to explain ERPs (lines 101-105): “ERPs are the average of the EEG data that is time-locked to specific events or stimuli, in this instance the presentation of a face. Task dependent cortical processing is reflected in characteristic features of the ERP waveform, commonly referred to as components (see Sur & Sinha, 2009 or Woodman, 2010 for an introduction to ERPs).”

3. Reviewer 2 noted two sentences in the introduction that were long and hard to follow.

These have been broken up into multiple sentences to facilitate easier reading.

Edited text (lines 119-127): “The current study aims to extend previous work investigating responses to cleft lip/palate that has provided evidence for differences in early perceptual and neural responses to infants with facial malformations. We do so by measuring visual and neural responses to upright and inverted healthy and cleft lip/palate infant faces in the same participants. Additionally, we investigate the impact of these facial malformations on both early stages of face processing (N170, P200) important for structural encoding *and* later stages of face processing (the late positive potential, LPP) that reflect affective processing and are important for evaluating the impact of the cleft lip/palate on subsequent visual evaluation.”

Edited text (lines 148-152): “Lastly, given that cleft lip/palate represents a deviation from normal facial appearance and is linked to atypical visual engagement, we expect to find differences in the visual scanning of cleft faces compared to healthy, normal faces. We predict adults will spend more time viewing the mouth region (at the cost of eye gaze/contact) for the cleft faces.”

4. Reviewer 2 queried whether there are ethical considerations with permission to use images.

All images were obtained via a google image search. As such, these images are already “published” on the internet. Images were not shown outside the lab for our study. Regarding permission to publish, we note that the faces depicted in figure 1 are composite images of multiple individuals. As such, these are not “real faces” - rather they are exemplar images to demonstrate the type of faces shown to participants. Because we created these composites ourselves, no permission is required for use.

Edited text (lines 184-186): “Example face images from each category are shown in Figure 1 (note that composite face exemplars were created to protect the identities depicted in the images used in the actual study).”

5. Reviewer 2 asked that we more clearly outline the statistical analyses used, particularly in reference to the cuteness ratings.

Because we use different analyses for each measured variable (cuteness ratings, eye tracking, ERPs), we feel it is clearer for the readers if we describe the statistical analyses employed at the start of each relevant results section rather than in a separate statistical analyses section.

Added text (lines 286-287): “Cuteness ratings were subjected to a paired-samples t-test to determine the impact of cleft lip/palate on perceived cuteness.”

6. Reviewer 2 requested we use the ‘±’ symbol to reflect standard deviations throughout the results section.

We have corrected all instances where this symbol was not used.

7. Reviewer 2 asked that we more clearly articulate the threshold for significance.

We have updated the statistical analysis text for the eye tracking and ERP sections to more clearly articulate our statistical thresholds.

Edited text: (lines 306-312): “These measures were subjected to separate multifactorial repeated-measures ANOVAs with *ROI* (eyes, mouth) and *palate* (cleft, normal) as within-subject factors. To account for the increase in family-wise error rate associated with running multiple statistical tests, we set our pre-test alpha at .025 (i.e., .05/2). All posthot tests were Bonferroni corrected using the p.adjust feature in R.”

Edited text: (lines 347-352): “Repeated measures ANOVA was used to evaluate the effects of the within-subject factors of the palate (cleft/normal) and inversion (upright/inverted) on each ERP component…To account for the increase in family-wise error rate associated with running multiple statistical tests, we set our pre-test alpha at .0125 (i.e., .05/4). All posthoc tests were Bonferroni corrected using the p.adjust feature in R.”

8. Reviewer 2 asked that we re-word our description of the palate x ROI interaction for the time to first fixation data.

We have rewritten this section of the manuscript to increase the clarity of reporting.

Edited text (lines 317-323): “Paired t-tests showed that the time to first fixation of the eye ROI was significantly slower (*t*(17) = 3.72, p = .002, M_cleft_ = 2.06 ± 0.88 sec, M_normal_ = 1.58 ± 0.49 sec) while time to first fixation of the mouth ROI was significantly faster (t(17) = -4.02, p < 0.001, M_cleft_ = 1.62 ± 0.65 sec, M_normal_ = 2.20 ± 0.83 sec) for the cleft palate than the normal palate faces. This finding suggests that visual attention is more readily captured by the mouth region, at the expense of the eyes, for infant faces with cleft lip/palate.”

9. Reviewer 2 requested that we explain the use of inverted faces earlier in the manuscript.

We have updated the introduction section of the manuscript to better explain why inverted faces were presented in the EEG study.

Added text (lines 129-135): “Disruptions to configural processing, such as face inversion reliably increase the magnitude of the N170, possibly reflecting the recruitment of additional cognitive mechanisms to process the face (e.g. Sadeh & Yovel, 2010). Because cleft lip/palate disrupts the normal infant facial configuration, we expect an increase in N170 magnitude compared to healthy infant faces. Inverting the face will disrupt configural processing of both cleft lip and healthy faces and should result in a similar increase in the N170 magnitude for both face types.”

10. Reviewer 2 requested that we better introduce the ERPs investigated in the introduction section of the revised manuscript.

We thank the reviewer for highlighting the additional clarity this would provide to readers. We have incorporated a basic description of each ERP component along with our hypotheses regarding the impact of cleft lip and palate on these neural responses in the introduction section of the revised manuscript.

Added/edited text (lines 123-148): “Additionally, we investigate the impact of these facial malformations on both early stages of face processing (N170, P200) important for structural encoding *and* later stages of face processing (the late positive potential, LPP) that reflect affective processing and are important for evaluating the impact of the cleft lip/palate on subsequent visual evaluation. The N170 is generally considered an index of face processing and is impacted by configural manipulations (e.g., Bentin et al., 1996; Eimer, 2000). Disruptions to configural processing, such as face inversion, reliably increase the magnitude of the N170, possibly reflecting the recruitment of additional cognitive mechanisms to process the face (e.g., Sadeh & Yovel, 2010). Because cleft lip/palate disrupts the normal infant facial configuration, we expect an increase in N170 magnitude compared to healthy infant faces. Inverting the face will disrupt configural processing of both cleft and healthy faces and should result in a similar increase in N170 magnitude for both face types. The P200 is generally thought to reflect the encoding of the second-order spatial relations of a face and is considered to be sensitive to the “typicality” of a face (Schweinberger & Neumann, 2016). Because they make infant faces less typical, we expect the presence of a cleft lip/palate to also modulate the amplitude of the P200 ERP component. The LPP is a positive ERP component beginning around 400ms after stimulus onset that is modulated by the motivational relevance of the stimuli (Schupp et al., 2000). It is evoked by emotionally engaging stimuli (Liu et al., 2012) and is thought to reflect an enhanced evaluation of salient visual stimuli (Hajcak et al., 2009). Because the LPP tends to be larger for negative stimuli (Glazer et al., 2018) and facial disfigurements have been shown to elicit negative emotional responses from observers (e.g., Macgregor, 1990; Partridge, 1998; Stone & Potton, 2014), we expect the LPP will be enhanced by cleft lip/palate.
