# Supplementary material for: Effects of cleft lip on visual scanning and neural processing of infant faces
Source: PLoS One. 2024 Mar 27;19(3):e0300673. doi: 10.1371/journal.pone.0300673 (PMC10971660; doi:10.1371/journal.pone.0300673)
Supplement: Attachment — Submitted filename: Response to Reviewers 1.docx [file pone.0300673.s003.docx]

**Journal Requirements/Editor’s Comments:**

1. The editor asked that we confirm our reference list is complete/correct.

We have confirmed our reference list is complete.

**Reviewer #1 Comments**

Reviewer 1 noted that we addressed all of their original comments and had only minor edits to request for our new submission.

1. Reviewer 1 asked that we further specify the definition of time to first fixation on lines 290-291.

Edited text (lines 289-291): “Time to first fixation (i.e., the interval between stimulus onset and the first gaze onset) was defined as the onset of the first gaze fixation in each ROI.”

2. Reviewer 1 asked that include further clarification regarding the number of statistical tests to better explain our corrected p-value thresholds (lines 296-297 & 329-330).

Edited text (lines 296-298): “To account for the increase in family-wise error rate associated with running statistical tests on the two measures, we set our pre-test alpha at .025 (i.e., .05/2).”

Edited text (lines 331-333): “To account for the increase in family-wise error rate associated with running statistical tests on the four ERP measures, we set our pre-test alpha at .0125 (i.e., .05/4).”

**Reviewer #2**

Reviewer 2 had no additional comments on the revised manuscript.
